# Training needs of informal caregivers of people with dementia in rural South western Uganda

**DOI:** 10.1002/alz70858_099133

**Published:** 2025-12-25

**Authors:** Joyce Namwase, Julius Kyomya, Amos Oyuru, Abraham Muhwezi, John Semuwemba, Emmanuel Maleka, Christine K Karungi, Celestino Obua, Edith Wakida

**Affiliations:** ^1^ Mbarara University of Science and Technology, Mbarara, Mbarara, Uganda

## Abstract

**Background:**

Dementia is a global public concern with increasing prevalence especially in low‐ and middle‐income countries. The burden associated with caring for people with dementia relies on informal caregivers who are usually family members. The aim of this study therefore was to identify the specific training needs of informal caregivers of patients with dementia in rural southwestern Uganda to better their caregiving roles and improve their own psychosocial health.

**Methods:**

We conducted 20 in‐depth interviews with 17 informal caregivers and 3 key informants who are workers from an elderly care home (Reach One Touch One Ministries) in Rukiga district, southwestern Uganda. The interviews were audio recorded and transcribed verbatim. A coding matrix was formulated and used to generate the appropriate codes from the transcripts.

**Results:**

The key findings from this study include caregivers' limited knowledge of dementia, with the condition being attributed to aging and stress. The informal caregivers face challenges such as physical, emotional, and financial burden due to the caregiving demands. The coping mechanisms included relying on faith, social support, and adapting their caregiving practices. Caregivers expressed a strong willingness to attend in‐person training sessions focusing on communication with people with dementia, managing dementia‐related behaviors, personal hygiene, and nutrition. The key informants highlighted the need for increased community awareness to address misconceptions about dementia and improve caregiving outcomes.

**Conclusion:**

Informal caregivers in rural Uganda face various challenges while caring for persons with dementia. Equipping them with skills in communication, dementia‐related behavior management, hygiene, and nutrition could enhance the quality of care for dementia patients while alleviating the physical and emotional burden on caregivers. Additionally, increasing community awareness to tackle misconceptions about dementia is essential for a supportive environment for caregivers and persons with dementia. These findings highlight the need for the integration of caregiver training and education into broader public health initiatives for dementia care.

**Key words**: Informal caregivers, Dementia, Training needs, Dementia caregivers, Rural Uganda

